# Sex Dimorphism Influences Cortical Microglial Morphological and Phenotypic Marker Profile after Closed Head Mild Traumatic Brain Injury in Rats

**DOI:** 10.1177/2689288X251377030

**Published:** 2025-09-11

**Authors:** Pooja M. Datta Roy, Jens Cuba, Kyle S. Milligan, Wenqi Shi, Dongmei Wang, Michelle C. LaPlaca

**Affiliations:** ^1^ Wallace H. Coulter Department of Biomedical Engineering, Georgia Institute of Technology and Emory University, Atlanta, Georgia, USA.; ^2^ Parker H. Petit Institute for Bioengineering and Bioscience, Georgia Institute of Technology, Atlanta, Georgia, USA.; ^3^ FAMU College of Engineering, Florida State University, Tallahassee, Florida, USA.; ^4^ School of Electrical and Computer Engineering, Georgia Institute of Technology, Atlanta, Georgia, USA.

**Keywords:** neuroinflammation, microglia functional state, mild traumatic brain injury, morphological analysis, sex dimorphism

## Abstract

Neuroinflammation is a nearly ubiquitous secondary injury process after traumatic brain injury (TBI) involving microglia. The time course of microglial functional transition between pro-inflammatory and anti-inflammatory states after mild TBI (mTBI) and the potential influence of sex in microglial response is not well-understood. To investigate interactions between sex and microglial activation states in the subacute post-mTBI period, we performed a morphological and phenotypic marker analysis on cells from male and female rats following closed head single impact (smTBI), repetitive impacts (rmTBI), or sham conditions at 24 h, 72 h, or 1 week postinjury. There was a significant increase in microglia population 24 h post-smTBI and at all time points for rmTBI in both male and female cells. Single-cell morphological analysis (24 microglia per animal) revealed no clear sex differences in microglial activation state. However, Sholl analysis demonstrated an increase in branching complexity for smTBI female cells at 24 h (area under the curve [AUC] 154 ± 2.1, *p* = 0.03) and at 72 h for rmTBI (AUC 229 ± 6.6, *p* = 0.006), but no increase in branching was observed in male cells. Principal component analysis similarly demonstrated that female cells formed distinct clusters at 72 h and 1 week, suggesting a change in morphology. There was an increase in anti-inflammatory marker, CD206, at 72 h for female cells in both smTBI and rmTBI groups. However, for males, most cells were KV1.3-positive (pro-inflammatory) even at 1 week in smTBI and rmTBI groups. Altogether, these data demonstrate microglial cells are pro-inflammatory 24 h after mTBI, but there is a robust difference between sexes, with female cells transitioning earlier from the pro-inflammatory state to the anti-inflammatory state compared with male cells. These results contribute to our understanding of sexual dimorphism associated with microglial recovery following mTBI and warrant further study of associated cellular pathways.

## Introduction

The recovery period and return to homeostatic function after mild traumatic brain injury (mTBI) is not fully understood due to the complexity and heterogeneity of persistent secondary injury cascades such as edema, cell death, and inflammation.^1–4^ Resolution of faulty post-TBI signaling is critical for optimal recovery. While most symptoms of mTBI resolve in days to weeks, subclinical cellular injury increases the risk of cognitive deficits, behavioral issues,^4^ and neurodegeneration.^5,6^ Currently, there are no pharmacologic therapies to prevent ongoing damage due to the host of interconnected cellular pathways. Therefore, targeting a single target may not be an effective approach, yet neuroinflammation may be a common pathway, and augmenting anti-inflammatory signals may have beneficial pleiotropic effects.^7–9^

Under homeostatic conditions, microglia are primarily responsible for immunosurveillance and initiation of inflammation in the brain.^5,10^ While microglial states are recognized to be fluid rather than binary,^11,12^ it is generally accepted that microglial cells have two main functional states after injury that exist on a continuum: pro-inflammatory and anti-inflammatory.^13,14^ Pro-inflammatory microglia are responsible for scavenging danger-associated molecular patterns (DAMPS), engulfing cellular debris, and recruiting peripheral immune cells.^15–17^ Cell morphology at this stage is ameboid with a low branching pattern.^13^ On the other hand, anti-inflammatory microglia exhibit hyper-ramified morphology^18^ and are responsible for quenching inflammation, tissue repair, and homeostatic maintenance.^19^ After injury, it is not clear whether pro-inflammatory or anti-inflammatory microglia predominate.^17,18,20–23^ Understanding this transition may further shed light on potential therapeutic targets that enhance anti-inflammatory-mediated recovery after mTBI.

The aim of this study was to quantitatively analyze the transition of microglial functional state after single impact mTBI (smTBI) or repetitive impact mTBI (rmTBI) in male and female cortical microglial cells. A subacute timeline (24 h, 72 h, and 1 week) corresponded to the dynamic microglial activation phase.^2,24^ The influence of sex on the microglial state transitions after injury is not consistent with many previous studies have reported conflicting results between male and female morphological changes,^25,26^ underlying the fact that microglial state is complex, nuanced, and influenced by many factors. For example, TBI has been shown to lead to a more robust neuroinflammatory profile in male cells versus female cells in mice after moderate and severe open head injury.^27–29^ Similar findings have been reported in an awake closed injury study.^30^ Other studies show no significant differences in microglial activation between males and females following experimental open head severe TBI.^31,32^ However, to our knowledge, there are paucity of studies reporting differences due to sex in microglial cells after a mild closed head injury, the most prevalent form of TBI. Thus, this study aims to further investigate potential sex differences in microglial activation by combining three-dimensional cell morphological analysis with inflammatory cell surface markers that represent cellular changes that occur after mTBI. It was hypothesized that both smTBI and rmTBI would elicit an acute inflammatory response to activate pro-inflammatory microglia, but the transition from a pro-inflammatory to an anti-inflammatory state would differ between male and female cells.

## Materials and Methods

### Animals

All procedures involving animals were performed according to the guidelines set forth in the Guide for the Care and Use of Laboratory Animals (U.S. Department of Health and Human Services, Pub no. 85-23, 1985) and were approved by the Georgia Institute of Technology Institutional Animal Care and Use Committee (protocol #A15088). Fischer rats (Charles River Laboratories; Wilmington, MA) were double housed in a 12:12 reversed light cycle and provided food and water *ad libitum*. Three groups (sham, smTBI, and rmTBI) were analyzed at three time points (24 h, 72 h, and 1 week postimpact). All tissue samples analyzed were derived from a previous independent original study (R21NS091832).

### Injury model

A single or repetitive closed head injury was delivered using a modified cortical controlled injury^9^ device (Pittsburgh Precision Instruments, Pittsburgh, PA, USA) with a 1-cm diameter silicone stopper (Renovators Supply Manufacturing, Erving, MA, USA) on the end of the standard CCI 5 mm piston. Rats were anesthetized with isoflurane (induction: 5%; maintenance: 3%) and placed prone on a 1 inch thick ethylene-vinyl acetate foam bed (McMaster-Carr, Elmhurst, IL, USA) and removed from anesthesia 1 min prior to closed head impacts similar to previously reported studies,^33,34^ in order to minimize potential anesthesia artifacts^35–39^ that may compromise preclinical TBI model fidelity.^40^ The impacts were delivered at the midpoint between bregma and lambda skull suture landmarks of the dorsal surface of the closed head at a velocity of 5 m/s (50 mins dwell time) with head displacement of 5 mm. Repetitive injuries consisted of two impacts 24 h apart. After the injury, the righting reflex latency was recorded. Mean righting reflex latency was 87.6 ± 21.9 sec (sham), 89.2 ± 55.5 sec (smTBI), and 94 ± 37.3 sec (rmTBI), indicating that the injury was mild.

### Tissue preparation

Sham and injured animals were sacrificed via transcardial perfusion (ice cold 0.1 M phosphate buffer, pH 7.4, followed by 4% paraformaldehyde in 0.1 M phosphate buffer) at 24 h, 72 h, or 1 week post-mTBI after the final impact. Brains were harvested, postfixed in 4% PFA for 24 h, cryopreserved in 30% sucrose, and coronally cryosectioned (20 µm).

### Morphology analysis

For morphological analysis, Gpower3.1 was used to calculate sample size using *a priori* power analysis using a two-tailed test.^41^ A medium effect size (f = 0.25), and an alpha of 0.05 was used. Results showed that a total sample of 372 cells with 18 equal sized groups was required to achieve a power of 0.95. We sampled 24 cells per animal from three tissue sections in both the right and left hemispheres of the dorsal cortex, directly under the impact site, for a total of 864 cells in 36 animals. Single cells were imaged at 40x magnification and captured using z-stack imaging (Zeiss LSM700, Oberkochen, Germany). FIJI-ImageJ (https://imagej.net/software/fiji/) was used to create a skeletonized graphic of the binary single-cell image, which was then processed by the skeleton plugin (Fiji-ImageJ) to detect circularity, cell area, span ratio, maximum branch length, branch number, sphericity, end-point voxels, junctions, triple point, and max span, similar to previous reports.^42–45^ See Supplementary Figure S1 for detailed descriptions of features. Sholl analysis was done to examine the complexity of branch ramification.^46^ Sholl analysis is a graphical tool that measures branching complexity by counting the number of intersections made by cell branches within a generated concentric field of circles from the center of the cell. Binary images were converted into an outline, and the Sholl plugin on FIJI-ImageJ was used to calculate the number of cell processes intersections. The more branches a cell has, the more interactions it will have with the concentric field of circles. The AUC scales with the number of interactions.

### Immunohistochemistry

For morphological analysis, all brain sections were immunostained with a general microglia/macrophage marker for ionized calcium-binding adaptor protein marker I (Iba1). Briefly, all sections were blocked in 0.03% Triton-X solution in PBS for 4 h at room temperature, followed by overnight incubation in primary antibody (anti-Iba1, 1:500, Wako 019-19741, Richmond, VA, RRID: AB_839504). The sections were then incubated in secondary antibody (goat anti-rabbit, 1:500, Thermo Fisher A11001, RRID: AB_2941012) for 4 h at room temperature and counterstained with DAPI (1:1,000, Invitrogen D1306, Waltham, MA, RRID: D1306) for 15 min before cover slipping. For functional state analysis, cells were colabeled with anti-Iba1 (1:500, Invitrogen MA5-27726, Waltham, MA, RRID: MA5-27726) and the pro-inflammatory marker KV1.3 (1:100, Alomone Lab APC-101, Jerusalem, Israel, RRID: APC-101)^47–49^ or anti-inflammatory marker CD206 (1:50, Invitrogen MA1-80069, Waltham, MA, RRID: MA1-80069).^13,50–54^ Antigen retrieval was performed by submerging slides into boiling 1x citric acid for 40 minutes prior to blocking. The remainder of the staining was performed as described above. Secondary antibodies used were goat anti-mouse (1:500, Thermo Fisher A11001, RRID: AB_2534069) and goat anti-rabbit (1:500, Thermo Fisher T-2767, RRID: AB_2556776), respectively. For morphological analysis, three brain sections were chosen at random along the anterior to posterior distance under the site of impact (bregma −3.1 to −4.6 mm). From each section, eight individual microglia were identified for a total of 24 microglia per brain (864 cells total). For phenotypic marker analysis, two brain sections near the dorsal cortex directly under the area of impact were chosen at random. Four areas of interest were identified in the cortex of each brain section to produce a total of eight images per animal. All cells that coexpressed Iba1 and DAPI were included in the analysis. Morphological, functional state immunostaining, and image analysis were done with relabeled slides such that the researcher was blinded to the condition and sex of the animal.

### Data analysis

All statistical analyses and graphs were generated using Prism GraphPad version 10 (GraphPad Software, San Diego, CA, USA). Sample distribution for cell counting, morphological analysis, and Sholl analysis was assumed to be normal, and a two-way analysis of variance (ANOVA) with Tukey’s multiple comparison *ad hoc* test was performed. For the functional state analysis, samples were analyzed using an ordinary one-way ANOVA parametric test with a Dunnett’s multiple comparison test. In addition, a nonparametric test (Kruskal–Wallis test with Dunn’s multiple comparison) was done for comparison, given that a normality test was not possible on the subgroup level.

## Results

### smTBI and rmTBI led to microglial activation and morphological changes

**FIG. 1.**
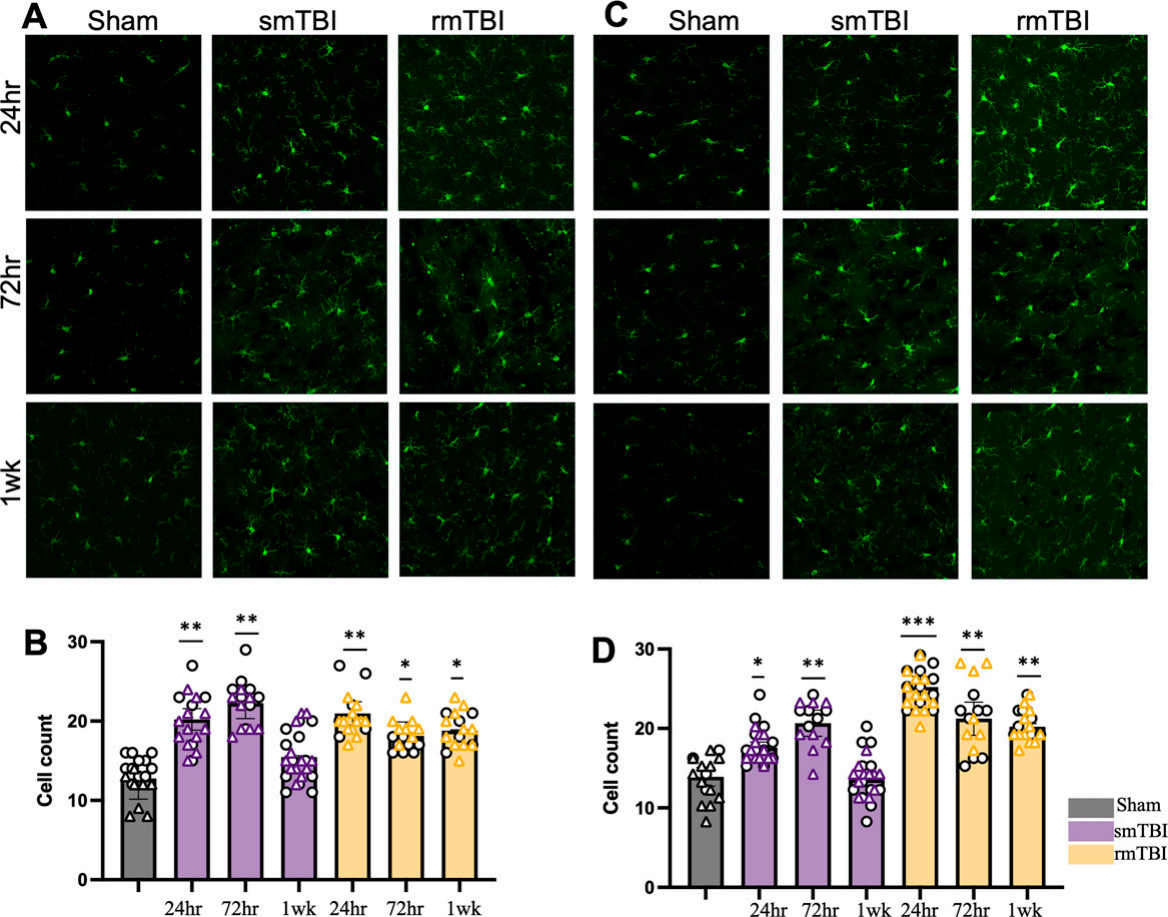
Microglia are activated following smTBI and rmTBI in both male and female brains. Microglial count increased after single and repetitive mTBI in males **(A** and **B)** and females **(C** and **D)** and remained elevated up to 1 week postinjury. *p* = *−< 0.05, **< 0.005, ***= 0.0005. rmTBI, repetitive impacts traumatic brain injury; smTBI, single impact TBI.

A significant increase in microglial population was observed after injury in male (Fig. 1A, B) and female cells (Fig. 1C, D) for both smTBI and rmTBI groups. In the smTBI group, for males, microglia cell count increased to 20.21 ± 1.36 in comparison to sham (12.57 ± 2.59, *p* < 0.05) at 24 h. In females, microglia cell count increased to 18.17 ± 1.53 in comparison to sham (13.92 ± 1.18, *p* < 0.05) at 24 h. Cell counts remained significantly elevated for both male cells (22.26 ± 1.95, *p* < 0.05) and female cells (20.67 ± 1.65, *p* < 0.05). However, at the 1 week time point, no significant differences were observed between the sham and injured groups in either male or female cells.

In the rmTBI condition, for males, microglia cell count increased to 20.96 ± 1.473 (*p* < 0.0005), and for females increased to 25 ± 1.355 (*p* < 0.005) at 24 h. The cell count remained significantly elevated at 72 h for males (18.13 ± 1.77, *p* < 0.05) and females (21.23 ± 2.09, *p* < 0.05) and continued to remain elevated at 1 week for male cells (18.76 ± 0.098, *p* < 0.05) and female cells (20.21 ± 0.413, *p* < 0.05). However, a two-way ANOVA revealed no statistically significant interaction between sex and number of impacts (F [3, 8] = 0.1574, *p* = 0.922).

**FIG. 2.**
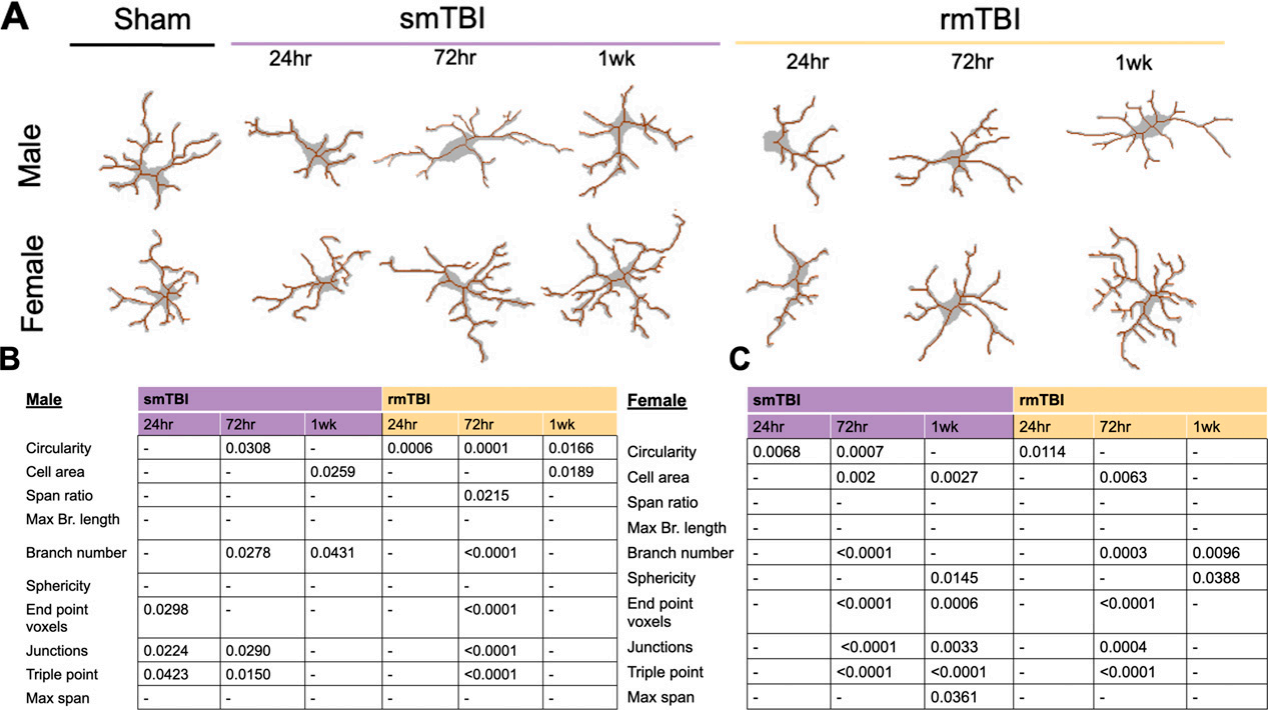
Table of microglial morphological characteristics in male and female brains following smTBI and rmTBI. **(A)** There is a visual difference in microglial morphology between different time points. **(B** and **C)** No pattern was identified between individual characteristics and functional activation state in males or females. rmTBI, repetitive impacts traumatic brain injury; smTBI, single impact TBI.

A binary and skeletonized mask of representative cells was created from each of the conditions for qualitative morphological comparison (Fig. 2A). Sham microglia overall had a circular outline and branches originating from the cell body for both male and female cells. Both sexes had similar morphological changes at 24 h postinjury, which included a loss of branching, decrease in cell body size, decrease in cell span, and increase in “rod-shaped appearance.” These characteristics appeared to be classical pro-inflammatory microglial morphology.^55^ However, at 72 h, a slight distinction between male and female cells started emerging. Male cells continued to express a low number of branching and an ameboid-like shape in the smTBI and rmTBI groups. However, more branching was observed in female cells in both injury groups. At 1 week, male cells also showed an increase in branching in the smTBI condition but remained unchanged in the rmTBI condition. In contrast, by 1 week, female microglia in both smTBI and rmTBI groups presented with a clear morphological change, which could be described as hyper-ramified. It could not be concluded whether these morphological changes were a pro-inflammatory phenotype or anti-inflammatory phenotype.

### Single-cell analysis was not sufficient to differentiate between activation state morphologies

Morphological characteristic change was quantified by using single-cell analysis in order to identify a pattern consistent with the phenotypic profiles. This was done using a single-cell analysis of individual cellular characteristics for both injury severities at 24 h, 72 h, and 1 week post-mTBI. Ten unique characteristics were identified based on previous studies^14,42–44,55^ including: circularity, cell area, span ratio, maximum branch length, branch number, sphericity, end-point voxels, junctions, triple point, and max span (Supplementary Fig. S1).

The overall trends in individual characteristic changes are summarized in Figure 2. In general, no clear pattern of characteristic changes was observed at 24 h, 72 h, or 1 week post-mTBI (Supplementary Figs. S2 and Supplementary Figure S3). However, certain characteristics relating to branching (such as branch number, junction, and end-point voxel) slightly increased at 72 h. These changes were not sustained at 1 week. Sholl analysis was done to further explore these observations.

**FIG. 3.**
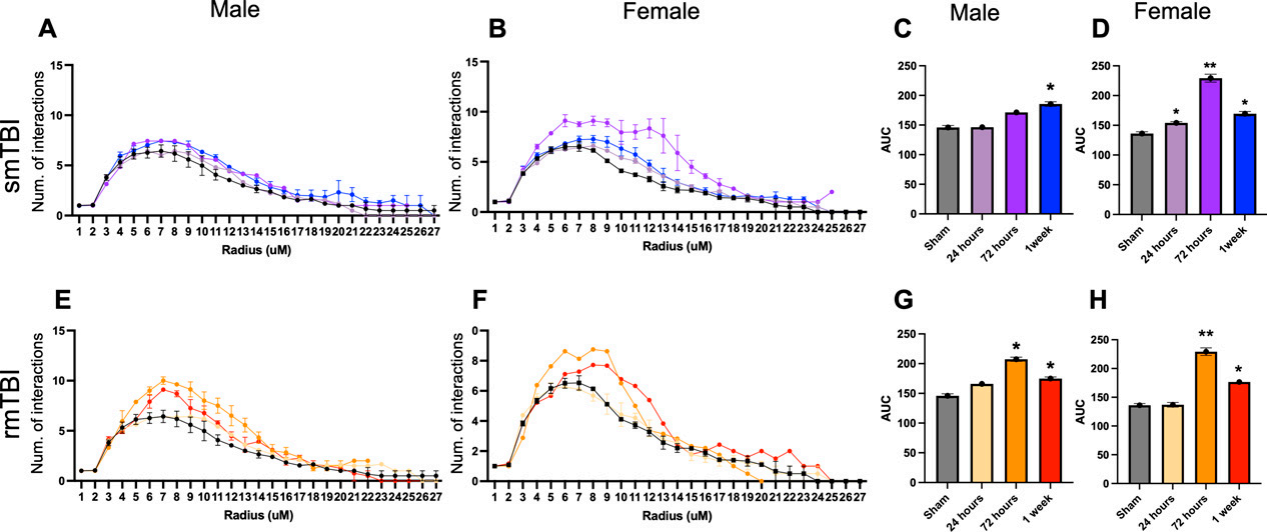
Morphological Sholl analysis of microglial cells in male and female brains after smTBI **(A** and **B)** and rmTBI **(E** and **F)**, respectively. Area under the curve (AUC) quantification shows that after smTBI, branching complexity increases after 1 week in males **(C)**, whereas an increase is evident by 24 h in females **(D)**. After rmTBI, branching complexity increases significantly after 72 h in males **(G)** females **(H)**. *p* = *<0.05, **<0.005. rmTBI, repetitive impacts traumatic brain injury; smTBI, single impact TBI.

Results from the Sholl analysis demonstrated that branching morphology changes with time after mTBI, number of impacts, and sex. The AUC for male cells increased at 1 week (185 ± 5.2, *p* < 0.05) and at 24 h for female cells (154 ± 2.1, *p* < 0.05) (Fig. 3A–D) in the smTBI group. In the rmTBI group, the AUC for male cells increased at 72 h (207 ± 5.1, *p* < 0.05) and remained elevated at 1 week. The AUC increased at 72 h for female cells (162 ± 5.2, *p* < 0.05) (Fig. 3E–H) and remained increased at 1 week in the rmTBI group. Together, these data show that microglia branching significantly increased sooner in smTBI (24 h) versus rmTBI (72 h). Furthermore, these data also demonstrate that an increase in microglial branching in female cells (24 h post-mTBI) occurs faster than in male cells (72 h post-mTBI).

### There is positive correlation between morphological features

Since no single morphological characteristic could be used to determine function, a correlation matrix was generated with the 10 characteristics to determine whether there were any interactions between the features under the various mTBI conditions. The correlation matrices revealed that many of the features were strongly correlated (Supplementary Fig. S4). For example, branch number, end-point voxel, junction, and triple point were highly correlated to each other. In contrast, span ratio, maximum branch length, and sphericity were negatively correlated to the other features. A principal component analysis (PCA) was done to investigate whether correlated features would create distinct clusters that could be used to visually segregate functional states.

**FIG. 4.**
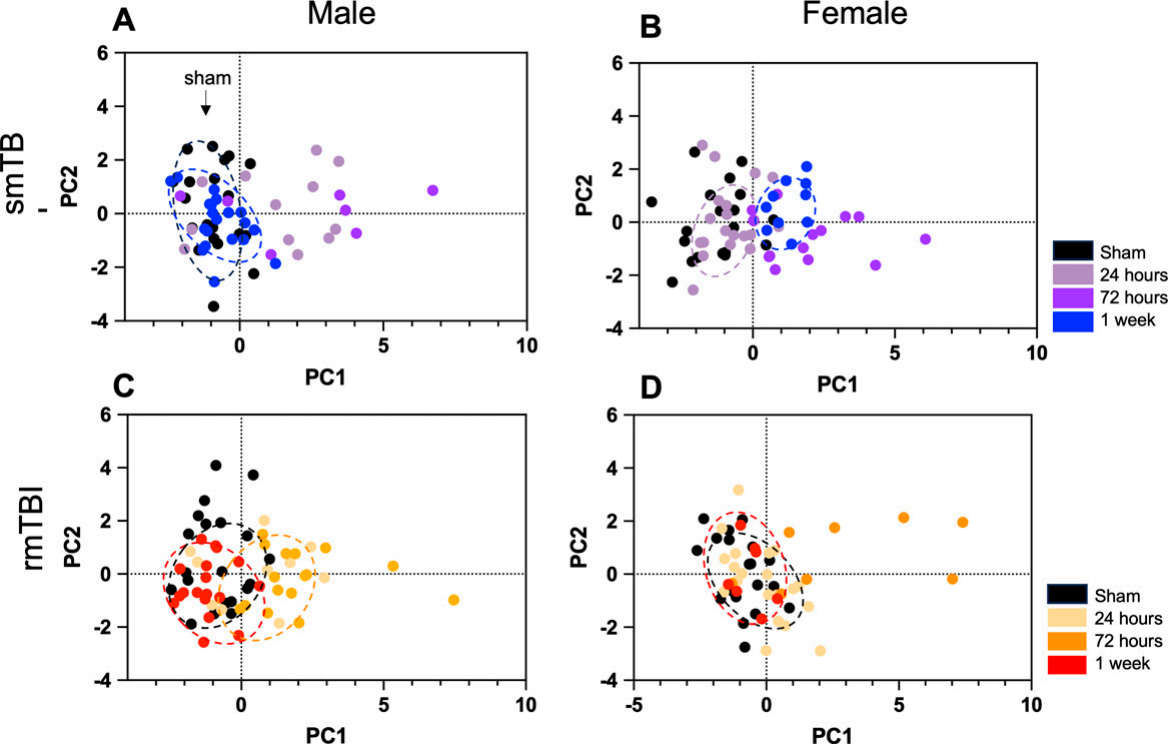
PCA plots showing clustering of functional activation states. Sham groups clustered left of the origin and possible anti-inflammatory groups clustered right of the origin. A pro-inflammatory cluster could not be identified. Male cells in the smTBI group **(A)** and rmTBI group **(C)** had a cluster overlapping the sham at 1 week, however the activation state of this cluster could not be identified. Female cells in the smTBI group **(B)** showed anti-inflammatory clustering at 1 week. This pattern was not observed in the rmTBI female cells **(D)**. PCA, principal component analysis; rmTBI, repetitive impacts traumatic brain injury; smTBI, single impact TBI.

**FIG. 5.**
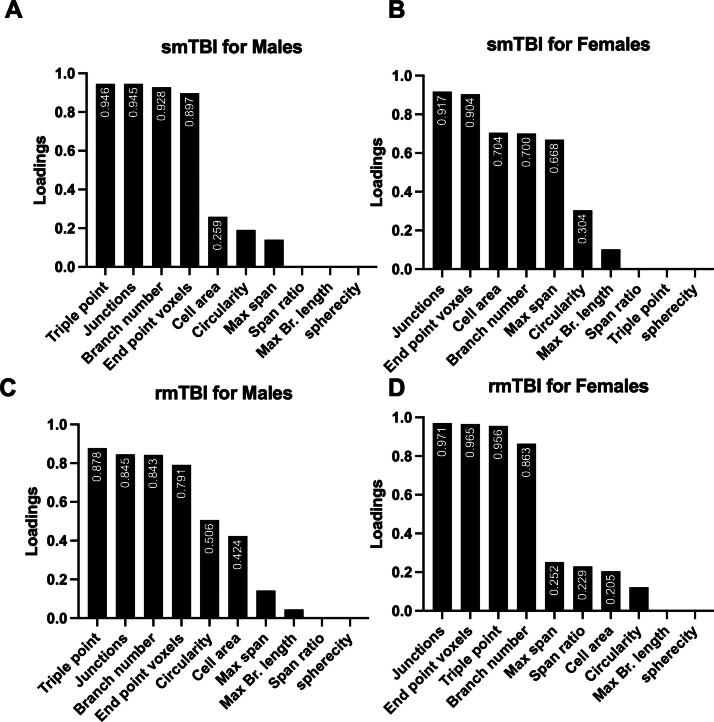
PCA loading plots. Of the 10 morphological features addressed in the PCA, four accounted for about 90% of the clustering: junctions, triple point, branch number, and end-point voxel. PCA, principal component analysis.

Distinct clusters were formed by the sham groups in all four groups (black dashed line), skewing left to the origin. In the smTBI group, males formed a distinct cluster after 1 week overlapping the sham cluster (Fig. 4A). Given that the Sholl analysis showed increased branching at this time point, it was difficult to determine if these cells were activated or had returned to homeostatic levels. Female cells formed one distinct cluster (Fig. 4B). Given that this cluster was formed by cells after 72 h, it may potentially represent cells in the anti-inflammatory phenotypic state. In the rmTBI group, male and female cells formed distinct clusters (Fig. 4C, D) at 72 h and 1 week. Based on Sholl analysis data, these groups may represent cells in a transitionary state or anti-inflammatory state. However, further investigation is needed to draw any conclusions. The PC loadings were examined to determine which morphological features contributed most to the clustering (Fig. 5). The loading tables show that ∼90% of the clustering was contributed by the following features: triple point, branch number, end-point voxel, and number of junctions.

After conducting PCA to identify potential clusters based on correlated features, t-distributed Stochastic Neighbor Embedding (t-SNE) was employed to further delineate and visualize the functional states in the dataset (Supplementary Fig. S5). t-SNE is a nonlinear dimensionality reduction method for embedding high-dimensional data into a space of two dimensions for visualizing complex, high-dimensional data. While typically used with subsets of data, t-SNE data analysis provided additional visualization that corroborated the PCA results.

The PCA suggested there was a transition from pro-inflammatory to anti-inflammatory state evident in female cells in both smTBI and rmTBI, but over different time courses. However, due to the fact that the overlapping clusters with sham could not be resolved, it was not evident whether there was a similar transition in male cells. To investigate this further, immunostaining using cell surface antibodies correlated to the pro-inflammatory and anti-inflammatory microglia states was conducted.

### Microglial costaining reveals distinctive activation states

Brain sections were colabeled with Iba1 and anti-KV1.3 (pro-inflammatory marker) or anti-CD206 (anti-inflammatory maker). Since Iba1 is a pan-macrophagic marker and cannot be exclusively used to distinguish between brain microglia and infiltrating peripheral macrophages, we are defining a pro-inflammatory state as Iba1 colabeled with KV1.3 and anti-inflammatory as Iba1 colabeled with CD206. Quantification of the colabeling was done by computing the ratio of KV1.3 positive (or CD206 positive) cells to Iba1 positive cells.

**FIG. 6.**
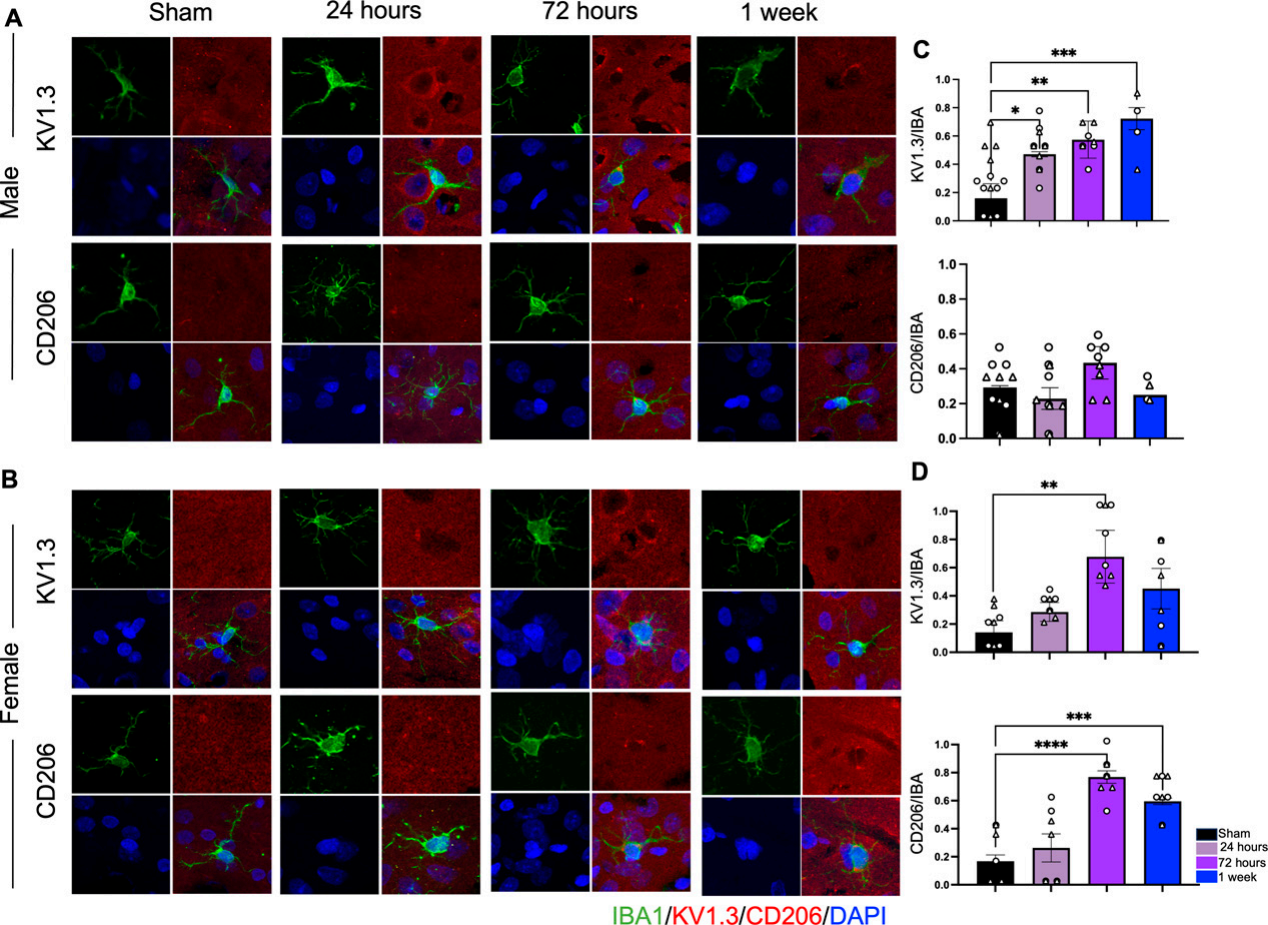
KV1.3 and CD206 expression in Iba1 + microglia cells in smTBI. **(A** and **B)** Immunohistological costaining of Iba1 + cells with KV1.3 and CD06 in male and female brains. **(C)** Quantification of immunostaining in males shows a significant increase in KV1.3 expression beginning at 24 h. However, there is no significant change in CD206 expression. **(D)** In contrast, in females, expression of KV1.3 was elevated at 72 h only. At the same time point, CD206 expression also increased and remained elevated until 1 week. *p* = *<0.05, **<0.005, ***<0.0005. smTBI, single impacts traumatic brain injury.

Male cells in the smTBI condition expressed higher KV1.3 than CD206 (Fig. 6, top 2 rows). In fact, expression of KV1.3 increased over time with the highest KV1.3/Iba ratio at 1 week (0.71 ± 0.08, *p* = 0.0009). In contrast, the CD206/Iba1 ratio did not significantly increase at any of the time points. For female cells in the smTBI group, a significant increase in KV1.3/Iba was seen at 72 h (0.68 ± 0.19, *p* < 0.05) (Fig. 6, bottom 2 rows). However, by 1 week post-TBI, there was no significant difference between sham and injured animals. A significant increase in CD206/Iba1 was also observed at 72 h (0.67 ± 0.09, *p* < 0.005) and remained elevated until 1 week (0.6 ± 0.02, *p* < 0.05).

**FIG. 7.**
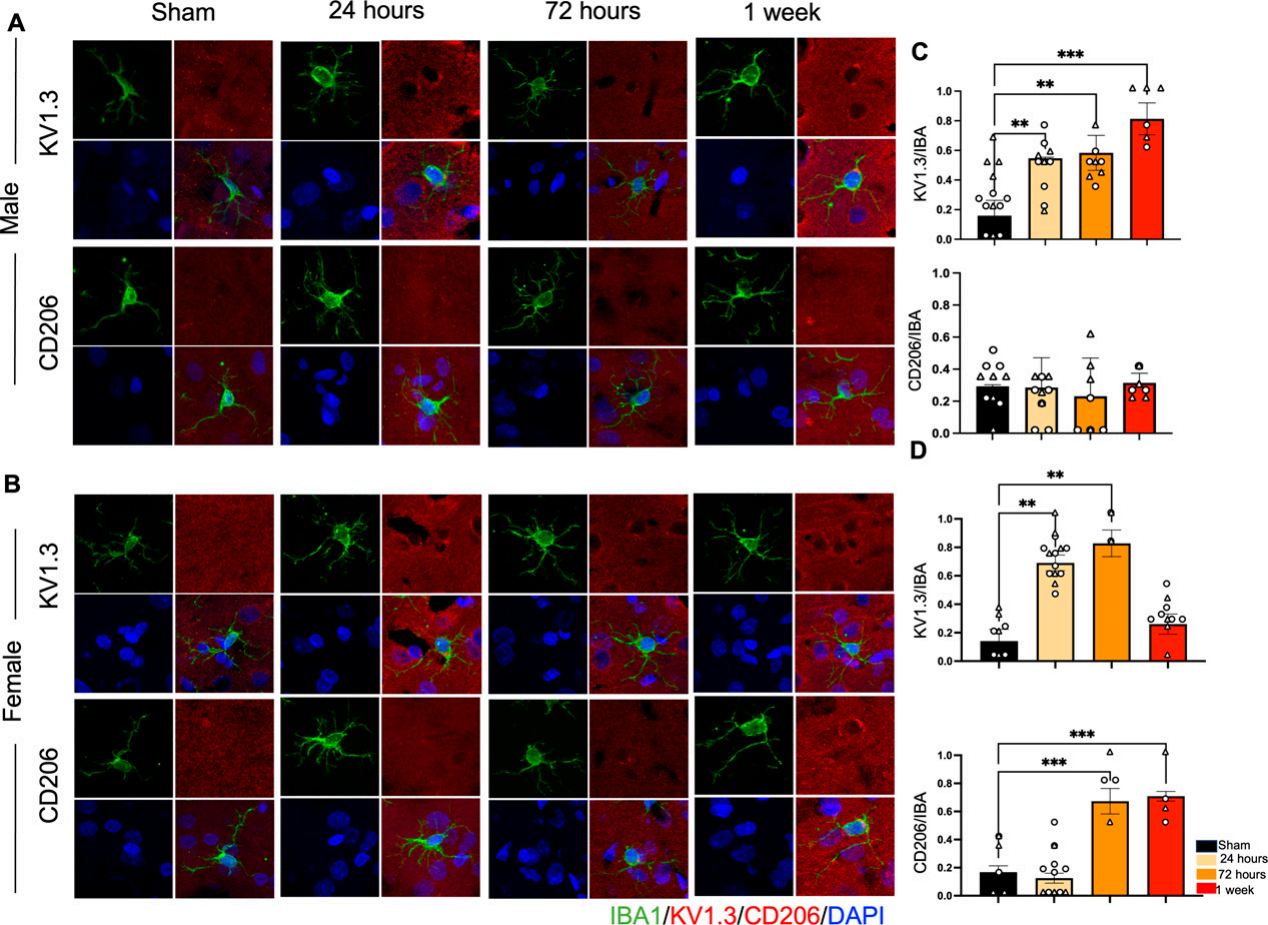
KV1.3 and CD206 expression in Iba1 + microglia cells in rmTBI. **(A** and **B)** Immunohistological costaining of Iba1 + cells with KV1.3 and CD06 in males and females. **(C)** Quantification of immunostaining in male brains shows a significant increase in KV1.3 expression beginning at 24 h. However, there is no significant change in CD206 expression. **(D)** In female brains, expression of KV1.3 increased by 24 h and remained significantly elevated until 72 h postinjury. At 72 h, CD206 expression also increased and remained elevated until 1 week. *p* = *<0.05, **<0.005, ***<0.0005. rmTBI, repetitive impacts traumatic brain injury.

Male cells in the rmTBI group also expressed high ratio of KV1.3/Iba, peaking at 1 week (0.81 ± 0.1, *p* < 0.005) (Fig. 7, top 2 rows). Expression of CD206/Iba was low in male cells and did not differ significantly from sham cells. For female cells in the rmTBI condition, KV1.3/Iba ratio was significantly higher at 24 h (0.69 ± 0.05, *p* < 0.05) and 72 h (0.83 ± 0.09, *p* < 0.05) but decreased by 1 week, at which there was no significant difference from sham (Fig. 7, bottom 2 rows). In contrast, at 72 h, CD206/Iba ratio increased (0.67 ± 0.09, *p* < 0.005) and remained elevated at 1 week post-TBI (0.71 ± 0.03, *p* < 0.0005).

Taken together, these results provide some interesting trends in microglia functional markers and transition after smTBI and rmTBI. It was observed that the pro-inflammatory functional state predominated after both smTBI and rmTBI. Interestingly, the transitional timeline differs between male cells and female cells. In male cells, there was as slight transition from pro-inflammatory to anti-inflammatory at 1 week in the smTBI condition. However, we did not observe any transition in the rmTBI condition. For female cells, the transition started as early as 24 h in the smTBI condition. However, it took up to 72 h in the rmTBI condition. Furthermore, there was a significant interaction between number of impacts and sex for the KV1.3 immunostaining [*F*(3,8) = 4.078, *p* = 0.049] and CD206 immunostaining [*F*(3,8) = 13.41, *p* = 0.0017]. These data present a substantial sex difference in post-mTBI neuroinflammation between males and females that was also dependent on the number of impacts.

## Discussion

In this study, we found that a pro-inflammatory phenotypic state is acutely present after mTBI, as expected. We also observed that female cells in the smTBI condition transitioned faster (24 h) from the pro-inflammatory state to an anti-inflammatory state in comparison to male cells in the same injury group (72 h). Similarly, female cells in the rmTBI condition transitioned faster (72 h) from the pro-inflammatory state to the anti-inflammatory state in comparison to male cells in the same group (>1 week). Within the same sex, female cells in the smTBI groups transitioned faster (24 h) than female cells in the rmTBI group (72 h). A similar effect was seen in male cells in the smTBI group that transitioned faster (72 h) than male cells in the rmTBI group (>1 week). Taken together, this study presents for the first time an interaction between the number of head impacts and biological sex on the transition time between activation states in microglia acutely following mTBI.

At 24 h postinjury in both smTBI and rmTBI groups, a large number of cells with the pro-inflammatory marker, KV1.3 were found colocalized with activated microglial cells. Furthermore, Sholl analysis at 24 h also displayed a lack of cellular branching further supporting the characteristic pro-inflammatory morphology. Although pro-inflammatory and anti-inflammatory functional states exist on a spectrum, these results indicate that the pro-inflammatory functional state predominates after rmTBI, similar to a previous study that also used a closed head injury model.^18^ In contrast, studies using an open head injury model have shown the anti-inflammatory functional state to be predominant in the subacute phase.^12,50,56^ This difference in functional state could be attributed to the fact that open head injuries are typically more severe than closed head injuries in animals and may be introducing additional confounds such as disruption of the blood–brain barrier,^57–59^ bleeding,^60^ and infiltration of peripheral immune cells.^61^ Another possible explanation for these observations is that the microglial anti-inflammatory function in more severe injuries may be augmented in order to reduce any further inflammatory and oxidative damage to the brain.^56^ In contrast, in closed head injuries, brain tissue may not be compromised to the same degree and microglial pro-inflammatory function may predominate to help engulf debris and recruit other immune cells.

In general, it took longer for the microglial cells to transition between the pro-inflammatory functional state and anti-inflammatory functional state in the rmTBI condition. In the Sholl analysis, this shift was identified based on the time it took for the AUC to significantly increase from the sham. In general, cells in the smTBI group made this transition within 24 h. However, it took up to 72 h for cells in the rmTBI condition. PCA supported these observations by the formation of distinct clusters representing resting state and anti-inflammatory states. The overall ratios of KV1.3/Iba1 and CD206/Iba1 were compared to determine which functional state predominated since microglia can express both pro-inflammatory and anti-inflammatory cell surface markers simultaneously.^12^ These results demonstrated that 24 h after injury, both male cells and female cells had a higher KV1.3/Iba1 than CD206/Iba1 indicating both were in the pro-inflammatory state. However, at 72 h, the expression of CD206 was upregulated in the female cells but remained suppressed in male cells. Given that female cells also had a high KV1.3 expression may indicate that microglia express both pro- and anti-inflammatory markers on the cell surface during the transitionary stage. An immediate increase in KV1.3 after injury and delayed increase in CD206 has been previously shown.^50,56,62,63^ Our research supports this finding but also shows that expression of these cell surface markers is dependent on both number of head impacts and sex.

A disparity in the results occurred between the Sholl analysis and immunostaining for male cells in the smTBI condition. For example, the AUC increased in the Sholl analysis at 72 h suggesting transition in functional state. However, there was no downregulation of KV1.3 expression or increase in CD206 expression that was expected for this transition state. Possible explanations for this may be that smTBI is too mild to elicit sufficient signals that promote reactivity, as well as individual animal variability that may diminish group differences even when sampling large numbers of cells.

The transition between pro-inflammatory and anti-inflammatory states could help guide timing for therapeutic interventions. Onset of microglia pro-inflammatory functional state can be attributed to wide range of cellular activities such as DAMPS production,^64^ reactive oxygen species generation and the presence of pro-inflammatory cytokines such as TNF-α, IFN-γ, and IL-6.^8^ In contrast, the microglia anti-inflammatory response is induced by anti-inflammatory cytokines such as IL-4 and IL-13.^65^ Signaling pathways such as TLR2,^66^ NfKB,^67,68^ STAT1 and STAT 3,^69^ STAT 6,^69^ and ROCK2^70^ have also been implicated in the transition between the microglial functional states. Furthermore, hormones such as progesterone may also be involved in the transition,^71,72^ supporting the observed difference between males and females. Studies have shown that progesterone is effective at blocking broad-spectrum potassium channels, including KV1.3,^73,74^ further explaining why female cells may have transitioned faster from the pro-inflammatory to the anti-inflammatory state.

One limitation of this study is a low animal subjects per group. However, the aim of this article was to study individual microglial morphology based on sex and injury severity. We believe we have analyzed a robust number of cells to make a correlation between our variables. Furthermore, similar studies that used morphological analysis reported low animal subjects per group, also focusing on multiple cells per brain for morphological analysis.^42–44^ Another limitation is the use of Iba1 as a microglial marker. Iba1 is a pan-macrophage marker that is used to identify peripheral macrophages as well as resident brain microglia. To address this issue, we triple-labeled with KV1.3 or CD206 and DAPI. Although KV1.3 and CD206 are not unique to microglial cells, these two markers are abundantly present on the microglial cell surface during activation.^22^ Thus, the presence of Iba1, an activation marker, and DAPI gave us confidence that we were identifying activated microglial cells. There was intentional heterogeneity introduced into the study design, with the variables of animal sex, injury severity, and time point, which can introduce additional variability, but is also necessary for robustness.^75^ Furthermore, the female estrous cycle was not accounted for, which may introduce more variability.^76^ However, it is recognized that females may not introduce as much variability as once assumed.^77^ We also did not discriminate between microglia in gray and white matter since we sampled from the dorsal cortex. There is a notable difference in cell surface markers and biochemical properties between microglial cells that reside in the white versus gray matter.^78,79^ The effect of these differences on morphology and function after injury would be interesting to evaluate in future studies. In sum, these data increase our understanding of sex differences in neuroinflammatory response to TBI that will need to be validated with a larger sample size in replicate studies.

## Conclusion

mTBI initiates a cascade of biological processes, including neuroinflammation. We showed that both single and repeat impacts elicit a morphological change in microglia causing them to activate towards the pro-inflammatory function. An overall faster change from pro-inflammatory to anti-inflammatory state following a single impact was observed, while cells from the repeat impact group required more time to transition. Furthermore, female cells made this transition faster in both conditions suggesting differential regulation of post-mTBI in female and male cells. Further investigation into the signaling pathways that mediate the transition between pro-inflammatory and anti-inflammatory activation states is warranted to identify potential therapeutic targets.

## Data Sharing

Data are available upon request to the corresponding author.

## Abbreviations Used

ANOVA: analysis of variance
AUC: area under the curve
CD206: Cluster of Differentiation 206
DAMPS: danger associated molecular patterns
DAPI: 4′,6-diamidino-2-phenylindole
Iba1: ionized calcium-binding adaptor protein marker I
IFN-γ: Interferon-gamma
IL-4: Interleukin-4
IL-6: Interleukin-6
IL-13: Interleukin-13
KV1.3: Potassium voltage-gated channel subfamily A member 3
mTBI: mild traumatic brain injury
NfKB: Nuclear factor kappa-light-chain-enhancer of activated B cells
PCA: principal component analysis
rmTBI: repetitive mild traumatic brain injury
ROCK2: Rho-associated coiled-coil containing protein kinase 2
smTBI: single mild traumatic brain injury
STAT1: Signal Transducer and Activator of Transcription 1
STAT3: Signal Transducer and Activator of Transcription 3
STAT6: Signal Transducer and Activator of Transcription 6
t-SNE: t-distributed Stochastic Neighbor Embedding
TBI: traumatic brain injury
TLR2: Toll-like receptor 2
TNF-α: Tumor Necrosis Factor alpha
