# Supplementary material for: Sex Dimorphism Influences Cortical Microglial Morphological and Phenotypic Marker Profile after Closed Head Mild Traumatic Brain Injury in Rats
Source: Neurotrauma Rep. 2025 Sep 11;6(1):790–803. doi: 10.1177/2689288X251377030 (PMC12528851; doi:10.1177/2689288X251377030)
Supplement: Supplementary Data [file 2689288x251377030_supplementary_data.docx]

Supplemental figure legends

**Supp Figure 1.** **Cell morphological features included in analysis.** Circularity: Overall roundness of cells. Cell area: negative space occupied by binary image. Span Ratio: ratio of the major over minor axes of the convex hull. Max branch length: length of longest branch. Branch number: Total number of branches and subbranches extending from cell center. Circularity: the radius ratio between the largest and smallest circumscribing spheres. End Point Voxel: voxels with less than two neighboring voxels. Junctions: intersection between branches. Triple Point: location where three branches meet. Max Span: longest diameter across the cell.

**Supp Figure 2.** **Single cell morphological analysis for male microglial cells**. In comparison to sham, most cell morphological characteristic did not show significant difference between time points under smTBI and rmTBI that corresponded to change in cell functionality. p: *<0.05, **=0.005, ***<0.0005, ****<0.00005

**Supp Figure 3.** **Single cell morphological analysis for female microglial cells**. In comparison to sham, most cell morphological characteristic did not show significant difference between time points under smTBI and rmTBI that corresponded to change in cell functionality. p *<0.05, **<0.005, ***<0.0005, ****<0.00005

**Supp Figure 4.** **Heat map of Pearson’s correlation matrix for microglia morphological characteristics.** Characteristics such as branch number, end point voxels, junctions, and triple point had a strong correlation (+0.5-+1.0) while characteristics such as span ratio, circularity and max span had a weak correlation (-0.5—1.0).

**Supp Figure 5.** **t-SNE plots illustrating the segregation of functional activation states.** Clusters with potential anti-inflammatory groups appear to be centered, while those associated with potential sham groups are scatted at the top and bottom. Notably, males in the smTBI group (A) demonstrated anti-inflammatory clustering at 72 hours, a pattern absent in the rmTBI male group (C). Females in both the smTBI (B) and rmTBI (D) groups exhibited anti-inflammatory clustering, observed at 1 week and 72 hours post-injury, aligning with PCA plot findings in Figure 3.
