# Supplementary figures and images for: Sex Dimorphism Influences Cortical Microglial Morphological and Phenotypic Marker Profile after Closed Head Mild Traumatic Brain Injury in Rats
Source: Neurotrauma Rep. 2025 Sep 11;6(1):790–803. doi: 10.1177/2689288X251377030 (PMC12528851; doi:10.1177/2689288X251377030)

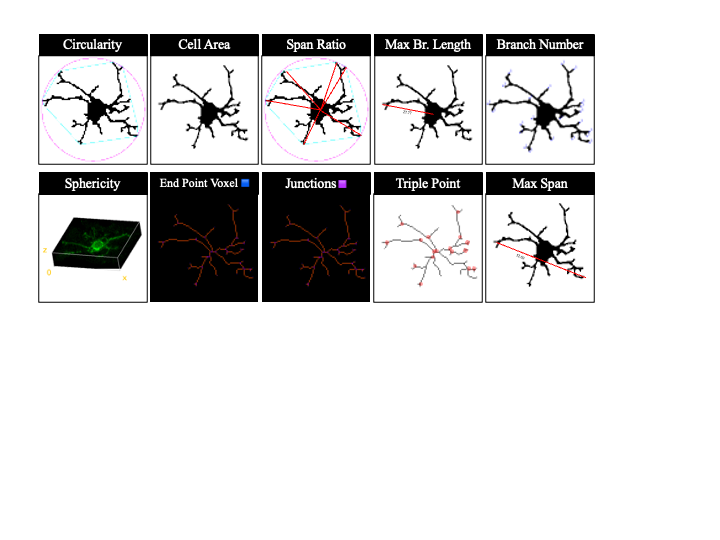

Supplement: Supplementary Figure S1 [file 2689288x251377030_supplementary_figure_s1.tif]

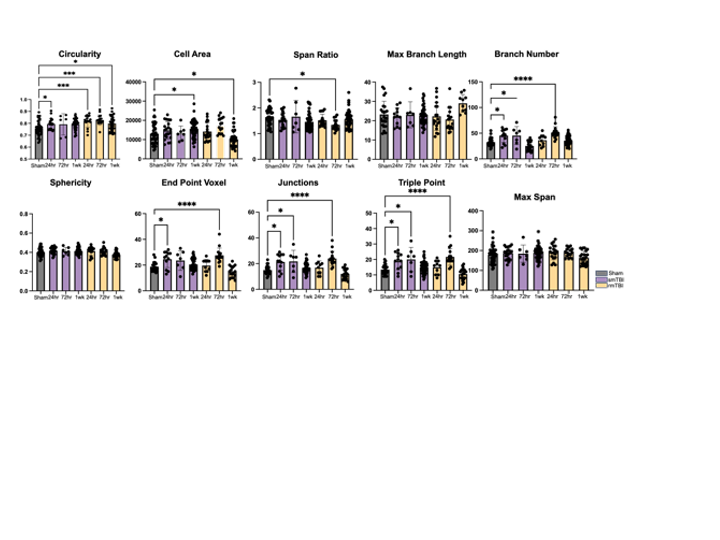

Supplement: Supplementary Figure S2 [file 2689288x251377030_supplementary_figure_s2.tif]

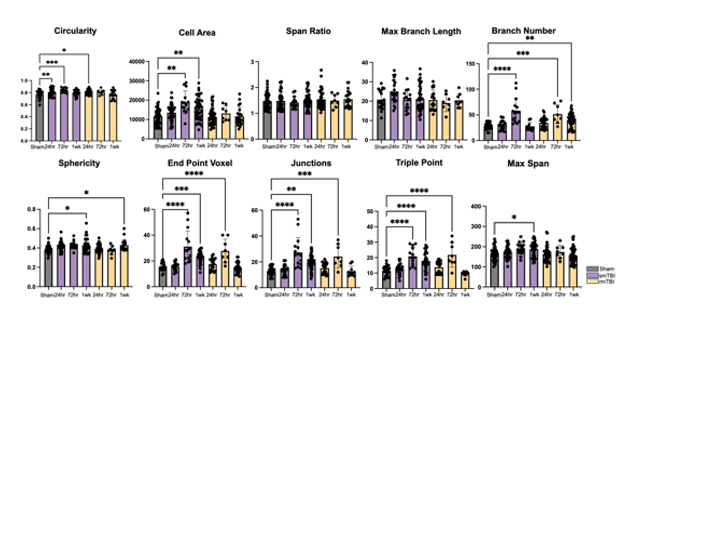

Supplement: Supplementary Figure S3 [file 2689288x251377030_supplementary_figure_s3.tif]

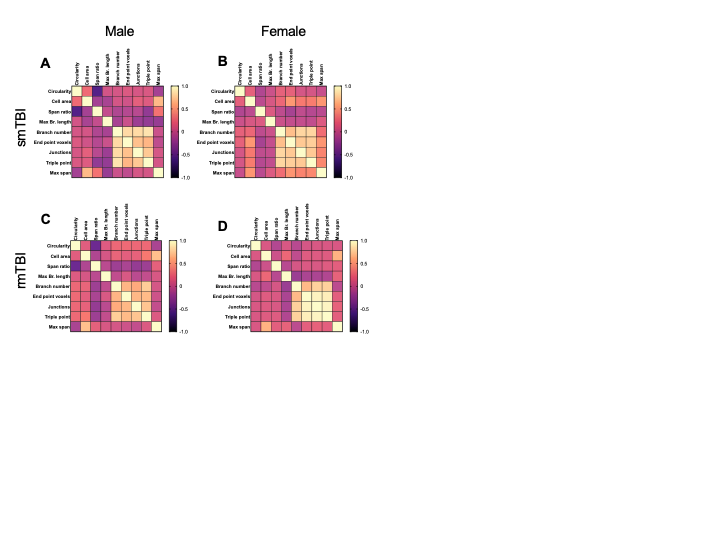

Supplement: Supplementary Figure S4 [file 2689288x251377030_supplementary_figure_s4.tif]

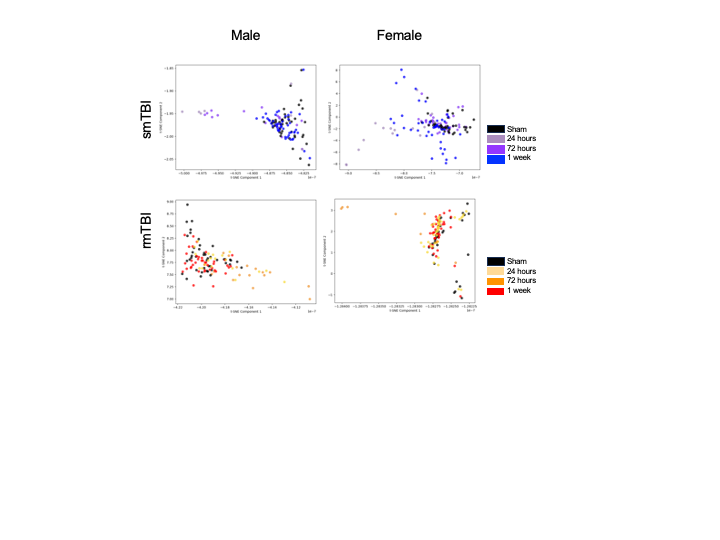

Supplement: Supplementary Figure S5 [file 2689288x251377030_supplementary_figure_s5.tif]
